# A Gap in the United States Healthcare System: Physician Nutrition Education Knowledge and Application

**DOI:** 10.15694/mep.2017.000193

**Published:** 2017-11-01

**Authors:** Kristen Hicks, Melody Howard, Peter Murano

**Affiliations:** 1 University of North Florida; 2 Texas A&M University

**Keywords:** medical education research, nutrition education, provider education

## Abstract

This article was migrated. The article was marked as recommended.

Physicians demonstrate an insufficiency in medical nutrition training, yet are expected to deliver nutrition counseling to patients with chronic disease. There is a clear understanding that unhealthy lifestyle behaviors (e.g. smoking, physical inactivity, poor diet) contribute to morbidity and mortality across the nation and worldwide. A preventable contribution to millions of deaths annually, which can be mitigated via brief nutrition and lifestyle counseling. Primary care is the ideal venue to deliver nutrition education and counseling, with a majority of all Americans regularly visiting their physician offices. With preventive medicine on the rise, is it imperative that a physician is proficient to have a sense of medical nutrition, to briefly counsel patients. This missing link, if fixed, will change the healthcare delivery system and overall patient outcomes for the better.

## Introduction

Physicians hold the most influence, among healthcare providers, when it comes to providing guidance regarding the general health and well-being of patients. Patient-physician relationships are directly associated with the patients’ behavior change. Thus, in order to prevent and/or manage chronic conditions, it is imperative that physicians develop positive and influential patient-physician relationships to assist with behavior change. According to the CDC 83.2% of all adults in the US had contact with their healthcare providers. ( CDC 2016) This capacity of physician-patient contact hours serves as premier opportunities for physicians to provide nutrition and lifestyle counseling to patients. Nationwide, physicians lack education in nutrition and lifestyle practices and report limited lifestyle counseling in practice with patients. Yet according to a 2011 survey, 64% of American’s believe physicians are “very credible” sources of nutrition information. ( Nutrition and You: Trends 2011 2011) There is irony present, in that physicians are sought to provide education, yet lack the education themselves.

For decades nutritionists and health care experts have expressed the need to increase the sheer number and availability of nutrition education programs for physicians. However, little progress has resulted. Survey results from US medical schools show that 71% do not provide the minimum hours of nutrition education (25 hours) and 36% provide less than half. ( Adams 2015) The driving need to develop guidelines and competencies to include lifestyle prescriptions and healthy behavioral modifications into practice has begun to be addressed. As part of the Healthy People 2020, several key objectives focus on increasing physician nutrition counseling for individuals with the modifiable chronic disease.(Healthy People 2020 2016) A recent systematic review recognized the disparity between lifestyle education and counseling delivery to patients and potential value (e.g. cost, morbidity) for preventing cardiovascular disease. ( Bock et al. 2012) The discrepancy between preventive care offerings such as nutrition and lifestyle education can be mitigated with more educational offerings for physicians.

It has been demonstrated that when nutrition education is delivered in primary care, it can modify risky behaviors of patients associated with chronic disease. ( Lin et al. 2014) A seven-year study conducted by primary care physicians performing monthly face-to-face lifestyle counseling resulted in a reduced frequency of hyperlipidemic, hyperglycemic and hypertensive periods. Moreover, a higher frequency of counseling sessions resulted in a greater number of patients who met lifestyle goals. ( Morrison et al. 2012) A systematic review of education delivered by a healthcare provider to cardiac patients also supports this theme, in that positive benefits related to physical activity, dietary intake and smoking cessation were observed. ( Ghisi et al., 2014) A retrospective study of over 10,000 hyperglycemic adults with diabetes mellitus documented that intensive lifestyle counseling was associated with an improved glycemic control. ( Hosomura et al. 2015) Similarly, the Diabetes Prevention Program (DPP) demonstrated that lifestyle intervention reduced type 2 diabetes mellitus diagnosis among high-risk patients by nearly 58%. ( Knowler WC. 2002; Dour 2013) Although the number of studies is relatively small, research studies that include physician-delivered nutrition and lifestyle education are consistently reporting improvements in clinical outcomes.

The message is robust: physician-delivered nutrition and lifestyle counseling is lacking, which hinders the potential to decrease chronic disease risk. When implemented, research shows physicians hold strong influence and stimulate change when it comes to the delivery of healthy behavior communication to patients. With disease and mortality on the rise in nutrition and lifestyle-related chronic disease, the time for physicians to be able to deliver lifestyle advice to patents could not be more urgent. Ultimately a shift to increase provider education will aid patients to positively impact nutrition and lifestyle factors.

## Take Home Messages

• Physician-delivered nutrition and lifestyle counseling is lacking nationwide, which hinders the potential to decrease chronic disease risk and improve patient health.
• An increase in nutrition education opportunities for healthcare providers will assist patients to positively change nutrition and lifestyle factors.

## Notes On Contributors

Kristen Hicks is an Assistant Professor at the University of North Florida, focused on researching methods of delivering nutrition education to physicians and the interdisciplinary team. She is also a practicing Registered Dietitian.

Melody Howard is an undergraduate student at Texas A&M University in Nutrition. She was previously mentored by Kristen Hicks during her doctoral degree and has interest in nutrition education for health professionals.

Peter Murano is an Associate Professor at Texas A&M University, focused on nutrition education for medical students and physicians. Previously Dr. Murano founded and ran an obesity institute, his research mission to understand and contribute to the field of research on nutrition and obesity.
