# Supplementary material for: A Gap in the United States Healthcare System: Physician Nutrition Education Knowledge and Application
Source: MedEdPublish (2016). 2017 Nov 1;6:193. [Version 1] doi: 10.15694/mep.2017.000193 (PMC10885313; doi:10.15694/mep.2017.000193)
Supplement: Peer review history

# Reviewer report: Reviewer response for version 1

**DOI:** 10.21956/mep.19383.r28403

**Published:** 2017-11-03

**Authors:** Subha Ramani

**Affiliations:** 1 Harvard Medical School, Brigham and Women's Hospital

This review has been migrated. The reviewer awarded 4 stars out of 5 I enjoyed reading this paper and the messages conveyed by the authors. They are absolutely correct in stating that nutrition education is important for clinical trainees since nutritional deficiencies and excesses are major contributors to chronic diseases that primary care physicians address frequently in their practices.The problem statement is well written, evidence from important literature has been summarized, and the impact on behavior change emphasized as it should.Though the authors have written about what they perceive as a local gap, this is a universal gap in medical education and I am certain medical educators around the world will agree.Curricula cannot just be didactic, they need to be integrated into patient communication and education in the real world. Access to healthy food among patients in lower socioeconomic groups is a barrier that needs to be tackled as well.

Reviewer Expertise:

NA

No decision status is available

# Reviewer report: Reviewer response for version 1

**DOI:** 10.21956/mep.19383.r28402

**Published:** 2017-11-02

**Authors:** BALAJI ARUMUGAM

**Affiliations:** 1 TAGORE MEDICAL COLLEGE AND HOSPITAL

This review has been migrated. The reviewer awarded 5 stars out of 5 I congratulate and appreciate the author for writing his personal view on physician nutrition education knowledge which is a very important aspect of any chronic disease management. The situation is still worse in the countries like India where we are bearing the double burden of under nutrition and over nutrition. Chronic diseases like Type 2 Diabetes in which diet management plays the major role in maintaining the glycemic control, the patients are mostly referred to dietitians. Several Indian surveys have found that physicians agree on the importance of nutrition in their medical practice but do not feel comfortable and adequately prepared to provide nutrition counseling to their patients. The nutrition education has to be a part of the medical curriculum and the physicians are to be trained properly. This article is an eye opener at this point of time.

Reviewer Expertise:

NA

No decision status is available

# Reviewer report: Reviewer response for version 1

**DOI:** 10.21956/mep.19383.r28401

**Published:** 2017-11-01

**Authors:** Trevor Gibbs

**Affiliations:** 1 AMEE

This review has been migrated. The reviewer awarded 4 stars out of 5 An interesting and important opinion piece, well structured, well researched and with a very positive message. Although the authors infer to a US issue, this is truly a global one that we all need to address. As someone who also supports the ideas of healthy lifestyle and healthy nutrition then I was in complete agreement with these authors thoughts. As always however, these words now need to be put into action and curricula developed on how to influence communities as to the values of a healthy diet- whatever that may be. It probably goes further than the immediate community in that policy change is also part of the answer. I look forward to reading further comments from these authors as to how they develop their ideas.

Reviewer Expertise:

NA

No decision status is available
